# Vaccine hesitancy and conspiracy theories: a Jungian perspective

**DOI:** 10.1192/j.eurpsy.2022.1276

**Published:** 2022-09-01

**Authors:** O.T. Khachouf, M. Saraga

**Affiliations:** 1 Lausanne University Hospital (CHUV), Dept. Of Psychiatry / Liaison Psychiatry, Lausanne, Switzerland; 2 Lausanne University Hospital (CHUV), Dept. Of Psychiatry / General Psychiatry, Prilly, Switzerland

**Keywords:** conspiracy theory, COVID19, vaccine skepticism, collective psyche

## Abstract

**Introduction:**

Endorsing conspiracy theories seems to constitute a major feature of contemporary collective anti-vaccine movements (Vignaud & Salvadori, 2019). As revealed by the COVID-19 pandemic, this contributes to increased worldwide vaccine hesitancy (de Figueiredo et al., 2020).

**Objectives:**

The present work aims at providing novel insight into the collective psychological underpinnings of conspiracy-based vaccine discourses.

**Methods:**

Our approach is inspired by Jung’s view that human groups produce narratives to project their collective conflicts (e.g., social, religious, political) onto reality. We analyze these projections in relation to the “halo effect” phenomenon, namely taking metaphorical extensions of (scientific) concepts at face value (e.g. Keller, 1995). Accordingly, we discuss one version of “the Great Reset” theory, claiming that COVID-19 vaccines are used by “the elite” to control behavior and abolish fundamental freedoms.

**Results:**

Our analysis suggests that Western societies are manifesting some of their existential concerns through anti-vaccine discourse. In “the Great Reset” narrative, *characters* (people, vaccines, elites, immune systems, etc.) and *plot* can be read as symbols of, respectively, *structural elements* of the collective psyche (socio-cultural values, aggressive drives, death anxiety, psychic defenses, etc.), and *dynamic interrelations* among these elements.

**Conclusions:**

Conspiracy theories can be understood as shared narratives serving the purpose of giving shape to collective fears. Within such a framework, references to “vaccines” and “immunity” are the manifestations of a state of crisis of collective psychic defenses.

**Disclosure:**

No significant relationships.

